# Mutations in the Reverse Transcriptase and Protease Genes of Human Immunodeficiency Virus-1 from Antiretroviral Naïve and Treated Pediatric Patients

**DOI:** 10.3390/v7020590

**Published:** 2015-02-10

**Authors:** Dinesh Bure, Muzamil A. Makhdoomi, Rakesh Lodha, Somi Sankaran Prakash, Rajesh Kumar, Hilal A. Parray, Ravinder Singh, Sushil K. Kabra, Kalpana Luthra

**Affiliations:** 1 Department of Biochemistry, All India Institute of Medical Sciences, New Delhi 110029, India; E-Mails: dinesh2141986@gmail.com (D.B.); muzamilbiochem@gmail.com (M.A.M.); sspkmc2k@yahoo.com (S.S.P.); rajeshkalra84@gmail.com (R.K.); hilalparray@gmail.com (H.A.P.); 2 Department of Pediatrics, New Delhi 110029, India; E-Mails: rlodha1661@gmail.com (R.L.); ravinder.rathore.singh@gmail.com (R.S.); skkabra@hotmail.com (S.K.K.)

**Keywords:** drug resistance, reverse transcriptase, protease, mutation, nucleoside RT inhibitors, non-nucleoside RT inhibitors

## Abstract

The success of highly active antiretroviral therapy (HAART) is challenged by the emergence of resistance-associated mutations in human immunodeficiency virus-1 (HIV-1). In this study, resistance associated mutations in the reverse transcriptase (RT) and protease (PR) genes in antiretroviral therapy (ART) naïve and treated HIV-1 infected pediatric patients from North India were evaluated. Genotyping was successfully performed in 46 patients (30 ART naive and 16 treated) for the RT gene and in 53 patients (27 ART naive and 26 treated) for PR gene and mutations were identified using Stanford HIV Drug Resistance Database. A major drug resistant mutation in RT gene, L74I (NRTI), and two such mutations, K101E and G190A (NNRTI), were observed in two ART naïve patients, while M184V was detected in two ART treated patients. Overall, major resistance associated mutations in RT gene were observed in nine (30%) and seven (36%) of ART naïve and treated children respectively. Minor mutations were identified in PR gene in five children. Few non-clade C viral strains (≈30%) were detected, although subtype C was most predominant. The screening of ART naïve children for mutations in HIV-1 RT and protease genes, before and after initiation of ART is desirable for drug efficacy and good prognosis.

## 1. Introduction

The introduction of highly active antiretroviral therapy has resulted in improved treatment outcome and survival rate in HIV-1 infected children [1]. The anti-HIV drugs that were initially developed to target clade B viruses, are also effective against non-clade B HIV including subtype C viruses [2,3,4,5,6]. The successful long term viral suppression by combination antiretroviral therapy and free of cost accessibility to it has led to a considerable increase in highly active antiretroviral therapy (HAART) usage in resource constrained settings like India. However, the emergence and spread of antiretroviral drug resistant HIV-1 genetic variants jeopardize the efforts to reduce the progression of HIV-1 disease and is one of the major factors responsible for therapeutic failure in HIV-1 infected children [7,8,9,10]. Further, the lack of proofreading activity of HIV-1 reverse transcriptase contributes majorly to the ability of the HIV-1 to generate a high degree of genetic variability [11].

Most of the current understanding about the genetic mutations in the viral genes selected by antiretroviral therapy, thereby leading to drug resistance is limited to studies on HIV-1 clade B infected patients. The information on genetic mutations responsible for antiretroviral therapy(ART) failure, in the subtype C viruses that are responsible for majority of the infections in India and sub-Saharan countries, is scanty, perhaps due to the relatively delayed accessibility of antiretroviral therapies in the developing countries. The high incidence of drug-selected mutations in HIV-1 has been demonstrated as a major cause of HAART failure in adults [7,8,9,12], however, there is still not much data available in children.

In an earlier study, baseline (transmitted) mutations were observed by us in the reverse transcriptase (RT) gene of HIV-1 from antiretroviral naïve adult patients [10]. The majority of the HIV-1 infection in the pediatric population is due to vertical transmission [13]. For successful outcome of antiretroviral treatment, it is important to screen the presence of baseline-transmitted mutations in HIV-1 from seropositive antiretroviral naïve children.

The existing studies reported in Indian HIV-1 infected children have addressed mutations in either the RT or protease (PR) genes [14,15], however, comprehensive data is not available on the mutations in both genes, in the antiretroviral naïve and ART treated children. This cross-sectional study was undertaken to assess the mutations in the RT and PR genes of HIV-1, in both antiretroviral naïve and treated HIV-1 infected Indian children.

## 2. Materials and Methods

### 2.1. Subject Selection

A total of 140 HIV-1 infected children (70 each of ART naïve and treated) who were diagnosed and managed as per the NACO guidelines [16], were recruited at the Pediatric Outpatient Department of the All India Institute of Medical Sciences (AIIMS), New Delhi, after obtaining written informed consent from their respective parent/guardian/Legally authorized representative. The All India Institute of Medical Sciences, New Delhi, is a referral center visited by patients from adjoining states of North India, in addition to those residing in New Delhi. Children in whom ART was indicated had received a combination of 2 NRTIs (Lamivudine with zidovudine or Stavudine) and 1 NNRTI (Nevirapine or Efavirenz) as per the national guidelines [16]. The demographic profile, clinical history and examination findings were recorded on a structured performa. This study was reviewed and approved by the Institutional Ethics Committee of AIIMS (IESC/T-335/2010).

### 2.2. Laboratory Measurements

The plasma viral load was determined by quantitative RT-PCR (Roche COBAS TaqMan HIV-1 v2.0; Roche Diagnostics, Indianapolis, IN, USA), according to the manufacturer’s instructions. Lower detection limit of the assay was 47 HIV-1 copies/mL. CD4^+^ T cell counts were estimated by flow cytometric analysis (BD Biosciences, Sparks, MD, USA) at the Department of Microbiology, AIIMS.

### 2.3. Drug Resistance Profiling

Blood samples of all the subjects were collected in EDTA vacutainers. HIV-1 proviral DNA was isolated from whole blood using QIAamp DNA Micro Kit (QIAGEN Ltd., Crawley, UK) following the manufacturer’s guidelines.

**Figure 1:**
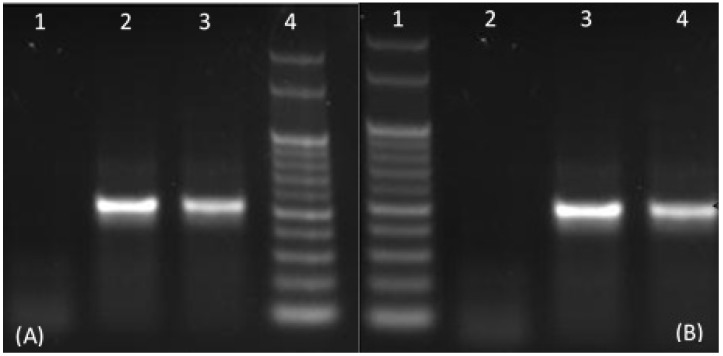
Representative gel picture of PCR amplified product of RT (**A**) and PR genes (**B**) of HIV-1 infected pediatric patient. For RT; Lane 1: Negative control, lane 2 and 3 contains 539 bp amplicon of RT gene of two representative HIV-1 infected children and lane 4:100 bp ladder. For PR; Lane 1: 100 bp DNA marker, lane 2: Negative control and 3 and 4: 514 bp amplicon of PR gene of two representative HIV-1 infected children.

HIV-1 RT and PR genes were amplified by nested polymerase chain reaction (PCR) using specific primers (Table 1). All amplifications were checked on a 1% agarose gel (Figure 1), stained with ethidium bromide and the DNA amplicons were purified using the QIAquick PCR purification kit (QIAGEN Ltd.) according to the manufacture’s details. The purified PCR products of both RT and PR genes were sequenced commercially. Mutations in the RT and PR genes responsible for resistance to ART were identified using the Stanford HIV Drug Resistance Database [17].

**Table 1:** List of primers for amplification of RT and PR gene.

*Primer*	*Orientation*	*HXB2 Position*	*Sequence*
Reverse Transcriptase			
RT1	Forward	2551–2572	5ʹ-TTCCCATTAGTCCTATTGAAACTGT -3ʹ
RT2	Reverse	3292–3313	5ʹ-TCATTGACAGTCCAGCTATCCTTT T -3ʹ
RT3	Forward	2705–2725	5ʹ-GCCTGAAAATCCATATAACAC TCC -3ʹ
RT4	Reverse	3219–3237	5ʹ-CCATCCAAAGAAATGGAGGTTC -3ʹ
Protease			
PR1	Forward	2082–2109	5ʹ-TAATTTTTTAGGGAAGATCTGGCCCTTC-3ʹ
PR2	Reverse	2734–2703	5ʹ-GCAAATACTGGAGTTGTATGGATTTTCAGG-3ʹ
PR3	Forward	2136–2162	5ʹ-TCAGAGCAGACCAGAGCCAACAGCCCC-3ʹ
PR4	Reverse	2650–2621	5ʹ-AATGCTTTTATTTTTTTCTTCTGTCAATGGC-3ʹ

Reverse and forward primers used for amplification of RT and PR gene of HIV-1.

### 2.4. Viral Subtyping and Phylogenetic Analysis

Sequences were analyzed for the subtype (clade) determination using REGA HIV-1 subtyping tool. Phylogenetic analysis of the RT and PR gene sequences was performed by including the HIV-1 reference sequences (from major subtypes (A-K) and other available circulating recombinant forms using Clustal X program. A phylogenetic tree was constructed by the neighbor-joining method by including 1000 bootstrap replicates using the Mega version 4 software [18].

### 2.5 Statistical Analysis

Data was recorded on a pre-designed worksheet and managed with MS office 2007. Data entry was double-checked. Statistical analysis was done using the intercooled STATA version 10.0 (STATA Corp., Houston, TX, USA). After confirming the normality aspects of the quantitative variables, the descriptive data was computed using mean and standard deviation.

## 3. Results

### 3.1. Patient Characteristics

A total of 140 HIV-1 infected pediatric patients were recruited after confirming their disease status as per the NACO guidelines (2006) [16]. All the HIV-1 infected children were managed as per the national treatment guidelines. Children on antiretroviral therapy (ART) were on a regimen of two NRTIs and one NNRTI as recommended by NACO [16]. None of the children were on protease treatment. Among the 140 HIV-1 infected children recruited for this study, 70 were antiretroviral naïve and 70 were on ART. DNA extraction was carried out in all the samples; further PCR amplification, DNA sequencing, viral clade determination and detection of the mutations was successful in 46 patients (30 drug naive and 16 drug treated) for RT gene and in 53 patients (27 drug naive and 26 drug treated) for the PR gene.

The demographic and clinical characteristics of the HIV-1 infected children are summarized in Table 2. The median age of antiretroviral naïve HIV-1 infected children was eight years (2–16) while that for the ART treated children was 10 years (2–17). Boys constituted 77% of the whole study population. The median CD4 counts were comparable between the two groups of infected children (*p* = 0.66), while the viral load was significantly lower in the ART treated children as compared to antiretroviral naïve (*p* = 0.02) (Table 2).

**Table 2:** Demographic and Clinical Data of HIV-1-Infected Indian Children.

Parameter	Naïve (*n* = 70)	Treated (*n* = 70)	*p*-Value
**Age** (Y), median (range)	8 (2–16)	10 (2–17)	0.06
**Sex**			0.55
Boys	52	56	
Girls	18	14	
**CD4 count**	645	769	0.66
cells/µL, median (range)	(17–2285)	(6–2269)	
**Viral load**	38,900	1780	0.02
RNA copies/mL, median (range)	(5190–228,000)	(47–38,200)	

### 3.2. Determination of Viral Subtype

**Figure 2:**
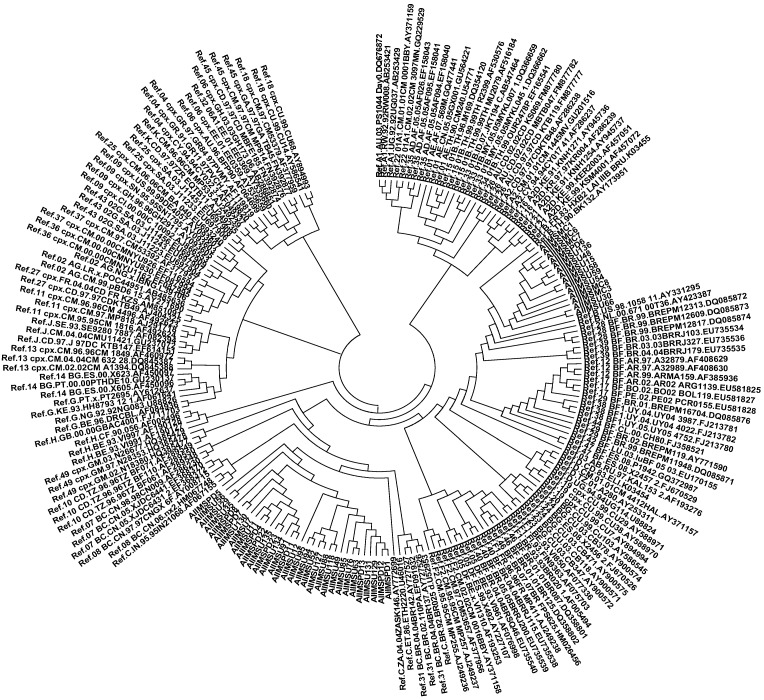
Neighbor-joining phylogenetic tree of Reverse Transcriptase sequences obtained from HIV-1 infected Indian children. A phylogenetic tree was constructed using MEGA 4.0 software using the neighbor joining method with 1000 bootstrap replicates. The sequences obtained from studied patients are labeled as AIIMS. 32 out of 46 RT sequences aligned with clade C while 14 aligned with subtype B sequences.

**Figure 3:**
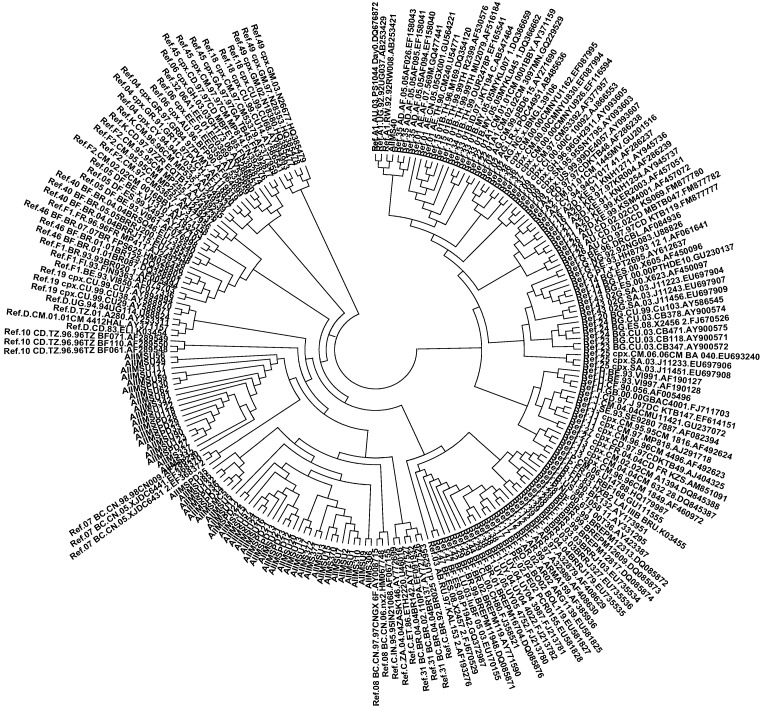
Neighbor-joining phylogenetic tree of Protease sequences obtained from HIV-1 infected Indian children. A phylogenetic tree was constructed using MEGA4.0 software using the neighbor joining method with 1000 bootstrap replicates. The sequences obtained from studied patients are labeled as AIIMS. 33 out of 53 PR sequences aligned with clade C while 19 aligned with subtype B sequences. PR sequences of AIIMS40 aligned with subtype A viral sequence.

The amplified PCR products of the RT (539 bp) and protease (514 bp) viral genes were purified and sequenced. Phylogenetic analysis for the RT gene revealed that 32 out of 46 (70%; Figure 2) sequences clustered with HIV-1 subtype C while 14 sequences aligned with subtype B sequences. Of the total fifty-three PR sequences, thirty-three (63%; Figure 3) clustered with HIV-1 subtype C, while 19 sequences (36%) aligned with subtype B sequences. Interestingly, the protease sequence from one antiretroviral naïve patient (AIIMSU40) aligned closely with a subtype A reference sequence.

The antiretroviral regimen for the HIV-1 infected pediatric group included a combination of two NRTIs (Lamivudine with zidovudine or Stavudine) and 1 NNRTI (Nevirapine or Efavirenz). In the present study, analysis of the viral RT gene sequences from the thirty ART naïve HIV-1 infected pediatric patients using the Stanford HIV drug resistance database revealed that nine (30%) had major drug resistance mutations for NRTI and NNRTI drugs (Table 3). Among ART treated pediatric patients, seven of the total nineteen (37%) harbored major drug resistance mutations for NRTI and NNRTI drugs (Table 3).

**Table 3:** Overview of the RT and PR resistance associated mutations in ART naïve and treated HIV-1 infected children.

Patient ID	CD4 Count (Cells/µL)	Viral Load (RNA Copies/mL)	Subtype	T/N ^#^	Major Drug Resistance Mutations		Minor Drug Resistance Mutations	Other Mutations *	Drug Regimen **	Duration of Treatment
Reverse Transcriptase					NRTI	NNRTI				
AIIMSU30	627	<47	B	T	D67E		L74Y	W88R, K102Q, L109Q, S162C, R206X, Q207X	NVP,LAM,STA	2 Years 8 months
AIIMSU35	837	1620	C	T	M184V	K103N		W88C, K101Q, D121H, K122E, I135L, K173A, Q174R, D177E, I178L, E194A, G196E, T200A, Q207E	NVP,LAM,STA	4 Years 1 month
AIIMSU48	569	NA	B	T		F227L		R206X, Q207X, L228X, M230E	LAM,STA,EFV	4 Years
AIIMSU52	288	NA	C	T	D67G		K65E	D121H, K122E, K173A, Q174R, D177E, T200A, Q207E, R211K	NVP,LAM,STA	4 months
AIIMSU56	698	27,500	C	T	M184V	K103N		K73X, I135T, D177E, I178L, T200A, Q207E, R211K, F214L, L228F, M230D	NVP,LAM,STA	6 Years
AIIMSU58	979	5570	B	N			K65E	K66E, R72E, L228F, M230N		
AIIMSU63	1528	34,200	C	N	L74I			R72K, D76X, D121Y, K122E, D123E, K173A, R206X, Q207X, L228X, M230G		
AIIMSU76	1459	1459	C	T	F77L			D76N, E79G, D121H, K122E, I135R, K173A, T200A, Q207E, R211K	NEV,LAM,ZDV	1 Year
AIIMSU85	1027	NA	C	N			L74Y	I63M, K64Q, T69X, R72K, K73i, I94K, D121Y, K122E, I135T, S162A, K173A, Q197K, T200A, R206X, Q207X, P226X, L228F		
AIIMSPD04	150	9480	C	N	K219Q			P55S, V60I, D121Y, K122E, S162A, K173A, D177E, T200A, Q207A, R211K, F214X, D218R, H221S		
AIIMSPD05	NA	<47	C	N		V106A		L109P, K122A, D123S, K173A, D177E, I178L, T200A, Q207X		
AIIMSPD11	1222	39,000	C	N	D67N	K101E, G190A		V60I, D121Y, K122E, I135K, S162A, K173A, D177E, T200A, Q207E, F214X		
AIIMSPD12	225	585,000	C	N		V179D		V60I, W88C, D121H, K122E, I135R, K173A, Q174R, D177E, T200A, I202V, Q207E		
AIIMSPD13	723	NA	B	N		F227L		K73X, D121H, K122E, S162T, L228F		
AIIMSPD16	NA	NA	B	N		V179F		D86H, G93 *, H96L, V106X, T107E, V118L, L120F, D121Q, D123Y, T128P, P133Q, I142M, Q145H, Q151R, E169Q, F171L, R206X, Q207X, W212R, P226A, Y232S, E233P, H235S, D237G		
**Protease**										
AIIMS306	1284	13,100	C	N			L10V	T12S, G16E, L19I, M36I, N37S, R41K, H69K, L89M, I93L		
AIIMSU40	225	24,000	A	T			L10I	I13V, E35D, M36I, N37E, R41K, R57K, H69K, L89M	NVP,LAM,STA	1 Year
AIIMSU59	1944	NA	B	T			I84T	E34Q, L63P	NVP,LAM,STA	2 Years 1 month
AIIMSU91	358	5190	B	N			L76T	L63P, K70N, G73N, V75P, V77I, T80P		
AIIMSPD27	679	NA	B	N			I84R	L63P, R87K, L97R		

This table includes only those patients who had mutations in the RT or PR gene. ^#^ T: ART treated, N: ART naïve; NA: Not available; * Mutations and/or polymorphisms that differ from a defined reference sequence/clade; ** NVP; Nevirapine, LAM; Lamivudine, STA; Stavudine, EFV; Efavirenz, ZDV: Zidovudine.

RT mutations conferring resistance to NRTI drugs were identified at positions 65,67,74,77,151,184,215,219 (Table 3). A salient observation of the study is the presence of the L74I mutation, which confers high level of resistance to didanosine, in a drug naïve patient (AIIMSU63). M184V mutation, which leads to high-level resistance to lamivudine and emtricitabine, was found in two ART treated children (AIIMSU35 and AIIMSU56). Two ART treated patients, AIIMSU30 (D67E and L74Y) and AIIMSU52 (K65E and D67G) had two mutations in the RT gene, known to confer resistance to NRTI drugs.

Mutations in the RT gene that confer resistance to NNRTI drugs were detected at amino acid positions 101, 106, 179, 190, and 227 (Table 3). Interestingly, the viral RT gene from an ART naïve patient (AIIMSPD11) had K101E and G190A double drug resistance mutations that are associated with high resistance to two commonly used NNRTI drugs, nevirapine and efavirenz. Several substitutions polymorphisms in the RT gene were found in nearly 30% of HIV-1 infected naïve children. The most frequent polymorphisms were seen at amino acid positions 60, 122, 162, 173, 177, 200, and 207 (Table 3).

Analysis of the PR gene sequence reveal that three ART naïve pediatric patients (AIIMS306, AIIMSU90 and AIIMSPD27) and two ART treated patients (AIIMSU40 and AIIMSU59) harbored minor drug resistance mutations, that could exert their effect only in combination with a major mutation (Table 3). Furthermore, no major drug resistant mutation in the HIV-1 protease gene was detected, from either naïve or ART treated pediatric patients.

## 4. Discussion

The incidence of HIV-1 infection in children has dramatically reduced in most of the developed countries due to prior administration of antiretroviral drugs to the mothers and their infants. A vast number of infants continue to acquire the infection in the developing countries of Africa and Asia including India due to limited access to ART. However, following the scaled-up measures including free and easy accessibility to ART, the HIV-1 incidence in children from the developing countries has shown an appreciable decline. Despite the successes, a major limitation associated with the antiretroviral therapy against HIV-1 is the emergence of genetic variants in the viruses selected by the ART drugs, which in turn can cause drug failure. This is a major public health concern, especially in children from resource-limited settings, owing to the limited monitoring options available and irregular and inadequate availability of ART. We have for the first time, assessed resistance associated mutations in the both the RT and Protease genes concurrently in naïve and ART treated HIV-1 infected children from India. Further, as the existing antiretroviral drug regimen for the HIV-1 infected children includes both 1^st^ and 2^nd^ line drugs, the mutation statuses of viral RT and PR genes have been assessed.

The low viral load in the ART treated patients as compared to the ART naïve children, observed in this study supports the effectiveness of antiretroviral drugs in controlling viremia, as has also been reported in earlier studies [13,19,20,21].

A major challenge for efficient clinical management of HIV-1 infected patients is posed by the presence of viral subtype diversity [22]. In the present cohort of HIV-1 infected pediatric patients, the presence of considerable number of subtype B viruses indicates the emergence of non-clade C viruses. The presence of BC recombinants in Indian HIV-1 infected adult patients has been recently observed [23,24], hence, emphasis should be on early subtyping in HIV-1 infected children, for the design of appropriate prevention strategies. There is an enhanced need for an early drug resistance genotyping in infants born to mothers who received suboptimal antiretroviral prophylaxis; due to the plausible vertical transmission of the drug resistant HIV-1 [25]. Limited information is available so far on the HIV-1 drug resistance in children from India and most of these are addressed in children on single dose drug therapy. Sehgal *et al.*, 2008, reported a high incidence of K103N mutation in ART naïve (33%) children and in those who had received a nevirapine containing regimen (56%) [14]. Another study on HIV-1 infected infants, on a single dose regimen of nevirapine, reported high levels of NNRTI mutation in samples collected at 48 hrs (10.5%) and 12 months (46.15%) respectively [14]. In the present study, the treated children were on HAART regimen that included a combination of two NRTIs and one NNRTI (NRTI: Lamivudine with zidovudine or Stavudine and NNRTI: Nevirapine or Efavirenz). An interesting and disquieting finding of this study is the high prevalence (30%) of major RT mutations conferring resistance to NRTI (23.33%) or NNRTI (16.66%) in ART naïve children, which in turn could be one of the potential factors that could lead to treatment failure. Two ART naïve patients, AIIMSU63 and AIIMSPD5 harbored L74I (conferring high level of resistance to didanosine; NNRTI inhibitor) and V106A (conferring high level of resistance to nevirapine; NNRTI inhibitors) mutations respectively. Also, K101E and G190A mutations, documented to cause high level resistance to nevirapine and efavirenz (NNRTI inhibitors), were detected in a naïve patient (AIIMSPD11). The presence of these major mutations that can confer resistance to the NRTI and NNRTI drugs, in ART naïve children, necessitates the screening of the ART naïve HIV-1 infected children for the presence of ART drug resistance mutations in the viral genes. The presence of major NRTI mutations in the ART treated children observed in this study underscores the need for a planned and judicious use of therapeutic regimens and emphasizes the need to undertake systematic drug resistance monitoring in HIV-1 infected individuals on HAART for a good prognosis.

Besides major drug resistant mutations, several polymorphisms were observed in the RT gene in nearly 30% of the HIV-1 infected antiretroviral naïve children, as has also been reported in earlier studies [10,26,27], suggesting some advantageous role of these polymorphisms in the pathogenesis of HIV [15].

The absence of any major mutation in the protease gene of the infected children in this study is in consonance with the previous reports on adults from India [28]. This can be explained by non-usage of PI in these patients. L10I is a polymorphic accessory mutation in the protease gene that is associated with reduced susceptibility to protease inhibitor [29]. The presence of L10I mutation in an ART naïve patient (AIIMSPDU40) is suggestive of the presence of such baseline transmitted mutations in the protease gene of HIV-1 in children, hence, screening for these mutations before initiating protease inhibitor treatment would be beneficial.

## 5. Conclusions

The presence of baseline (transmitted) and/or acquired mutations in the RT and PR genes of antiretroviral naïve and treated HIV-1 infected children, as observed in this study, suggests screening for the presence of resistance-associated mutations before and after the initiation of ART, for desirable drug efficacy and good prognosis. The rise in the emergence of clade B HIV-1 in Indian HIV-1 infected children must be accounted for while designing preventive strategies.
